# Comparison of two single-pill dual combination antihypertensive therapies in Chinese patients: a randomized, controlled trial

**DOI:** 10.1186/s12916-023-03244-4

**Published:** 2024-01-24

**Authors:** Qi-Fang Huang, Di Zhang, Yihong Luo, Kun Hu, Qiong Wu, Hailong Qiu, Fei Xu, Mei-Ling Wang, Xin Chen, Yan Li, Ji-Guang Wang, on behalf of the ACEI/CCB Versus ACEI/DIU Combination Antihypertensive Therapy in Chinese Hypertensive Patients (ACvAD) investigators

**Affiliations:** 1 Department of Cardiovascular Medicine, Centre for Epidemiological Studies and Clinical Trials, State Key Laboratory of Medical Genomics, Shanghai Key Laboratory of Hypertension, Department of Hypertension, The Shanghai Institute of Hypertension, Ruijin Hospital, Shanghai Jiaotong University School of Medicine, Ruijin 2nd Road 197, Shanghai, 200025 China; 2 Department of Cardiology, Chongming Branch of Shanghai Tenth People’s Hospital, Shanghai, China; 3 Department of Cardiology, Longhua Hospital, Shanghai University of Traditional Chinese Medicine, Shanghai, China; 4 Department of Cardiology, Suzhou Hospital of Anhui Medical University (Suzhou Municipal Hospital of Anhui Province), Suzhou, Anhui Province China; 5 Hypertension Center, Puyang People’s Hospital, Puyang, Henan Province China; 6 Department of Hypertension, Ruijin Hospital North, Shanghai Jiaotong University School of Medicine, Shanghai, China

**Keywords:** Single-pill combination, Antihypertensive, Amlodipine/benazepril, Benazepril/hydrochlorothiazide

## Abstract

**Background:**

Current hypertension guidelines recommend combination of an angiotensin-converting enzyme inhibitor or angiotensin-receptor blocker with a calcium-channel blocker or thiazide diuretic as initial antihypertensive therapy in patients with monotherapy uncontrolled hypertension. However, to what extent these two different combinations are comparable in blood pressure (BP)-lowering efficacy and safety remains under investigation, especially in the Chinese population. We investigated the BP-lowering efficacy and safety of the amlodipine/benazepril and benazepril/hydrochlorothiazide dual therapies in Chinese patients.

**Methods:**

In a multi-center, randomized, actively controlled, parallel-group trial, we enrolled patients with stage 1 or 2 hypertension from July 2018 to June 2021 in 20 hospitals and community health centers across China. Of the 894 screened patients, 560 eligible patients were randomly assigned to amlodipine/benazepril 5/10 mg (*n* = 282) or benazepril/hydrochlorothiazide 10/12.5 mg (*n* = 278), with 213 and 212 patients, respectively, who completed the study and had a valid repeat ambulatory BP recording during follow-up and were included in the efficacy analysis. The primary outcome was the change from baseline to 24 weeks of treatment in 24-h ambulatory systolic BP. Adverse events including symptoms and clinically significant changes in physical examinations and laboratory findings were recorded for safety analysis.

**Results:**

In the efficacy analysis (*n* = 425), the primary outcome, 24-h ambulatory systolic BP reduction, was − 13.8 ± 1.2 mmHg in the amlodipine/benazepril group and − 12.3 ± 1.2 mmHg in the benazepril/hydrochlorothiazide group, with a between-group difference of − 1.51 (*p* = 0.36) mmHg. The between-group differences for major secondary outcomes were − 1.47 (*p* = 0.18) in 24-h diastolic BP, − 2.86 (*p* = 0.13) and − 2.74 (*p* = 0.03) in daytime systolic and diastolic BP, and − 0.45 (*p* = 0.82) and − 0.93 (*p* = 0.44) in nighttime systolic and diastolic BP. In the safety analysis (*n* = 560), the incidence rate of dry cough was significantly lower in the amlodipine/benazepril group than in the benazepril/hydrochlorothiazide group (5.3% vs 10.1%, *p* = 0.04).

**Conclusions:**

The amlodipine/benazepril and benazepril/hydrochlorothiazide dual therapies were comparable in ambulatory systolic BP lowering. The former combination, compared with the latter, had a greater BP-lowering effect in the daytime and a lower incidence rate of dry cough.

**Trial registration:**

ClinicalTrials.gov, NCT03682692. Registered on 18 September 2018.

**Supplementary Information:**

The online version contains supplementary material available at 10.1186/s12916-023-03244-4.

## Background

Current hypertension guidelines recommend combination of an angiotensin-converting enzyme (ACE) inhibitor or angiotensin-receptor blocker with a calcium-channel blocker (CCB) or a thiazide diuretic as initial antihypertensive therapy in patients with monotherapy uncontrolled hypertension [1–5]. These combinations have been believed to be pharmacologically most appropriate because they are probably additive in the blood pressure lowering effect [6, 7] and counter-regulative in some of the side effects [8]. ACE inhibitors and angiotensin-receptor blockers combat the vascular and cardiac effects of the renin–angiotensin–aldosterone system by reducing and inhibiting angiotensin II [9]. CCBs dilate arterials and reduce peripheral resistance [10]. Diuretics reduce volume by increasing urinary excretion of sodium and water [11]. These drugs reduce blood pressure via different mechanisms and may also induce various adverse reactions, such as dry cough with ACE inhibitors [12], ankle edema with CCBs [13], and hypokalemia with thiazide diuretics [14]. There is some evidence that CCBs might reduce the incidence of dry cough induced by ACE inhibitors [15], and ACE inhibitors might reduce the incidence rate of ankle edema [13]. The combination of ACE inhibitors or angiotensin-receptor blockers with thiazide diuretics might also help in the maintenance of potassium homeostasis and reduce the risk of hyper- or hypokalemia [16, 17].

Although both combinations are pharmacologically appropriate and recommended as preferred therapy by several hypertension guidelines, these two combinations can still be clinically different, especially when several major demographic and clinical characteristics of patients are taken into account. Indeed, in a recently published randomized controlled trial in patients enrolled from several African countries, combination antihypertensive therapy that contained a CCB, either with an ACE inhibitor or a diuretic, was significantly more efficacious in lowering blood pressure than a combination of an ACE inhibitor with a thiazide diuretic [18]. The recently published International Society of Hypertension (ISH) 2020 guidelines recommend the preferred use of ACE inhibitors or angiotensin-receptor blockers with CCBs in the management of hypertension [3] on the basis of the results of the Anglo-Scandinavian Cardiac Outcome Trial (ASCOT) [19] and the Avoiding Cardiovascular Events through Combination Therapy in Patients Living with Systolic Hypertension (ACCOMPLISH) trial [20]. The latter trial compared the ACE inhibitor benazepril with the CCB amlodipine or hydrochlorothiazide combination and demonstrated that the benazepril and amlodipine combination was superior to the benazepril and hydrochlorothiazide combination in the prevention of cardiovascular events [20].

In China, ACE inhibitors, angiotensin-receptor blockers, and CCBs are the most frequently prescribed antihypertensive drugs, either as monotherapy or in combination. We hypothesize that the combination of an ACE inhibitor with a CCB, compared with the combination of an ACE inhibitor with a thiazide diuretic, is more efficacious in blood pressure lowering and has fewer side effects in the treatment of hypertension. The present randomized controlled trial was therefore designed to compare the 24-week treatment of amlodipine besylate 5 mg/benazepril 10 mg and benazepril 10 mg/hydrochlorothiazide 12.5 mg, both in single-pill combination (SPC), in blood pressure lowering and the incidence of side effects in the Chinese patients with stage 1 and 2 hypertension.

## Methods

### General design

The present study was a multi-center, randomized, actively controlled, parallel-group trial (ClinicalTrials.gov identifier number, NCT03682692) for the comparison between amlodipine besylate 5 mg/benazepril 10 mg (amlodipine/benazepril group) and benazepril 10 mg/hydrochlorothiazide 12.5 mg (benazepril/hydrochlorothiazide group) in patients with stage 1 or 2 hypertension from July 2018 to June 2021 in 20 hospitals and community health centers across China. The study protocol (Additional file 1) was approved by the ethics committee of Ruijin Hospital, Shanghai Jiaotong University School of Medicine, Shanghai, China, and, as necessary, also by the ethics committees of the participating hospitals. All patients gave written informed consent.

The study consisted of a 4-week benazepril-treatment run-in period and a subsequent 24-week randomized treatment period. If at a screening visit, previously untreated patients or previously treated patients on antihypertensive monotherapy had a blood pressure of 140–179 mmHg systolic or 90–109 mmHg diastolic and were willing to discontinue previous antihypertensive therapy, they entered the run-in period with antihypertensive monotherapy of benazepril 10 mg per day for the determination of eligibility. If eligible according to the average of six blood pressure readings obtained at two clinic visits 2 weeks apart and the tolerability to the benazepril monotherapy during the run-in period, patients were randomly assigned to receive amlodipine/benazepril one tablet per day or benazepril/hydrochlorothiazide one tablet per day for 24 weeks after stratification for study center. The study medication could be stopped in the presence of symptomatic hypotension or any other serious adverse events related to the study medication. Patients were instructed to take the study medication from 06:00 to 08:00 every morning before breakfast. If clinic systolic/diastolic blood pressure could not be controlled to the target (< 140/90 mmHg) during follow-up, the study medication could be up-titrated to two tablets per day. If there was no compelling indication, other antihypertensive agents or drugs of potential blood pressure-lowering action should not be used during the 24-week study treatment period. The study medication was supplied free of charge for the whole study period.

### Study participants

Men and women of 18 to 75 years of age were eligible for the trial, if the following inclusion and exclusion criteria were fulfilled. The average of the six clinic blood pressure readings measured at two clinic visits during the run-in phase with benazepril 10 mg per day was in the range of 140–179 mmHg systolic or 90–109 mmHg diastolic. No intolerable dry cough occurred with the benazepril monotherapy. The patients were able to attend the clinic visit on his/her own.

The exclusion criteria included the presence of life-threatening diseases, secondary hypertension, coronary heart disease, myocardial infarction, heart failure, stroke or dementia, abnormal liver (serum liver enzymes alanine aminotransferase or aspartate aminotransferase ≥ twice of the upper limit) and renal function (serum creatinine ≥ 1.5 mg/dL [133 µmol/L] or proteinuria on a dipstick test), the presence of contraindications to dihydropyridine CCBs or diuretics, and current participation in another trial.

Diabetes mellitus, defined as a plasma glucose of at least 7.0 mmol/L fasting or as the use of antidiabetic agents, was not an exclusion criterion. The estimated glomerular filtration rate (eGFR) was calculated from serum creatinine by the use of the Chronic Kidney Disease Epidemiology Collaboration (CKD-EPI) equation [21]. Chronic kidney disease was defined as an eGFR less than 60 mL/min/1.73 m^2^.

### Randomization

After 4 weeks of run-in period, eligible patients were randomized to the two study groups in a 1:1 ratio. Using the SAS software, an individual study statistician generated the randomization table with a block of four after stratification for the study center. The study investigators and coordinators were kept blinded before randomization. After the investigators, who enrolled the patients, sent the copy of the randomization tables to the coordinating center, one staff in the coordinating center assigned the randomization number and the treatment group according to the randomization table by center and table sequence.

### Randomized antihypertensive treatment and follow-up

Patients were randomly assigned to amlodipine 5 mg/benazepril 10 mg one tablet per day or to benazepril 10 mg/hydrochlorothiazide 12.5 mg one tablet per day. During each follow-up visit, the study medication could be up-titrated to amlodipine/benazepril or benazepril/hydrochlorothiazide two tablets per day to control clinic systolic/diastolic blood pressure to a level below 140/90 mmHg.

After randomization, patients were followed up every 4 weeks on normal working days in the morning. The follow-up time of the day was recorded. Clinic blood pressure and pulse rate were measured at baseline and at each of the follow-up visits. Ambulatory blood pressure monitoring was performed at baseline and at the end of the 24-week follow-up. Home blood pressure monitoring was performed for 7 days before each clinic visit at randomization and during follow-up. The responsible physicians collected information on the use of medications, adverse events, and serious adverse events at each follow-up visit. Blood and urinary biochemical measurements were performed at baseline and 4 and 24 weeks of follow-up. Hypercholesterolemia was defined as serum total cholesterol ≥ 5.18 mmol/L. Hypertriglyceridemia was defined as serum triglycerides ≥ 1.70 mmol/L. Dyslipidemia was defined as serum total cholesterol ≥ 5.18 mmol/L and/or serum triglycerides ≥ 1.70 mmol/L.

### Clinic, ambulatory, and home blood pressure measurements

Clinic blood pressure was measured three times consecutively with a 30–60 s interval after at least 5 min rest in the sitting position using a validated automated blood pressure monitor (HEM 9200T, Omron Healthcare, Kyoto, Japan). These three blood pressure readings were averaged for the clinical decisions at each follow-up visit and for the present analysis.

Ambulatory blood pressure monitoring was performed using an oscillometric ambulatory blood pressure monitor (TM2430, A&D Co., Ltd., Tokyo, Japan), which was programmed to obtain ambulatory blood pressure readings at 20-min intervals in the day (06:00–22:00) and at 30-min intervals at night (22:00–06:00). Daytime and nighttime were defined as the short clock time intervals from 08:00 to 18:00 and from 23:00 to 05:00, respectively. Valid recordings should cover more than 20 h and include at least 10 and five readings in the daytime and nighttime, respectively. The 24-h mean values of blood pressure and pulse rate were weighted for the time interval between consecutive readings.

Home blood pressure monitoring was performed using the same device as clinic blood pressure measurement (HEM 9200T, Omron Healthcare, Kyoto, Japan). Patients were provided with an Omron HEM 9200T monitor and requested to measure their blood pressure at home for seven consecutive days before each clinic visit at randomization and during follow-up. During the 7 days of home blood pressure monitoring, patients were asked to measure blood pressure three times in the morning before breakfast and three times in the evening at least 2 h after supper in the sitting position. Valid home blood pressure recordings should cover at least 5 days within a week, both in the morning and evening.

For clinic, ambulatory, and home blood pressure measurements, a standard cuff was used when the arm circumference was 32 cm or smaller. Otherwise, a large cuff was used.

### Efficacy and safety evaluations

The primary outcome was the change from baseline to 24 weeks of treatment in 24-h ambulatory systolic blood pressure. Secondary outcomes included the change from baseline to 24 weeks of treatment in 24-h ambulatory diastolic blood pressure, daytime and nighttime systolic and diastolic blood pressure, and clinic and home systolic and diastolic blood pressure.

All adverse events were documented for information on symptoms, severity, treatment, and outcome. The routine and biochemical tests of blood and urine were performed for clinical laboratory safety evaluations. Any clinically significant changes in physical examinations and laboratory findings were also recorded as adverse events.

### Sample size calculation

Assuming that the difference of 24-h ambulatory systolic blood pressure change after 24 weeks of treatment between the two groups is 2.5 mmHg and the standard deviation is 10 mmHg, *α* is 0.05, and the power is 80%, the sample size of each group should be 252 patients. The actual number of patients randomized in this study is 560. Among 560 eligible patients randomly assigned to amlodipine/benazepril 5/10 mg (*n* = 282) or benazepril/hydrochlorothiazide 10/12.5 mg (*n* = 278), 213 and 212 patients, respectively, who completed the study and had a valid repeat ambulatory BP recording during follow-up and were included in the efficacy analysis.

### Statistical analysis

Data management and statistical analysis were performed using the SAS software, version 9.4 (SAS Institute, Cary, NC, USA). The efficacy analysis was performed in the patients who completed the 24-week study and had a valid repeat 24-h ambulatory blood pressure recording at 24 weeks of follow-up. The safety analysis was performed on all patients who had ever started the study medication. Continuous and categorical variables were compared using the Student *t*-test and *X*^2^ test, respectively. Blood pressure and pulse rate changes from baseline were calculated by subtracting the values at baseline from those during follow-up. Analysis of covariance was performed to calculate the least square mean changes with standard error and the between-group mean difference with 95% confidence intervals (CI) with baseline values as covariate and treatment group as a factor. We also applied generalized estimating equation (GEE) analysis to compare blood pressure-lowering efficacy between the groups by considering the center as a possible bias.

## Results

### Patient characteristics at baseline

**Fig. 1:**
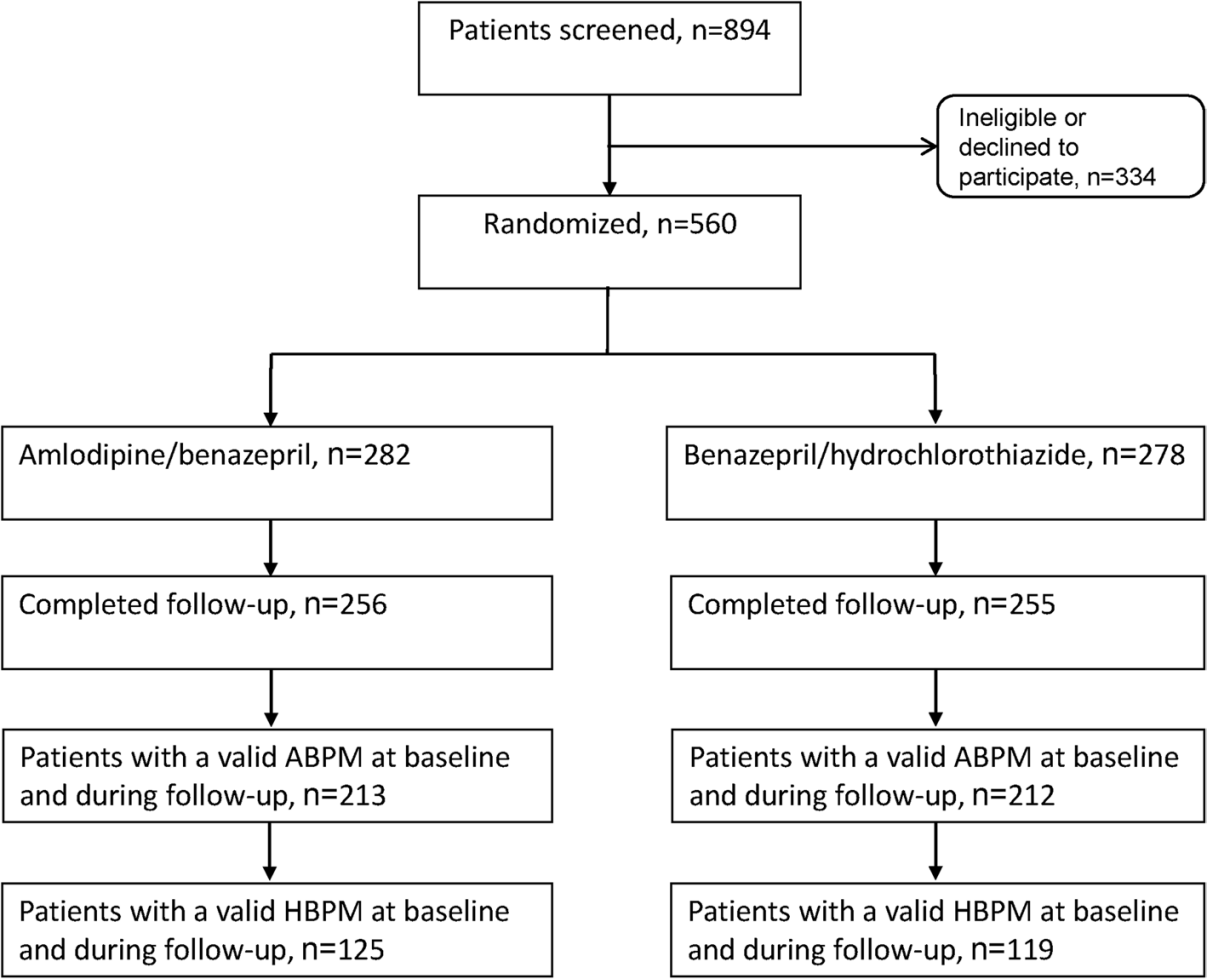
Flow of patients. ABPM, ambulatory blood pressure monitoring; HBPM, home blood pressure monitoring

Of the 894 screened patients, 334 were excluded because of ineligible blood pressure (*n* = 77), intolerable cough (*n* = 12), hyperuricemia (*n* = 8), proteinuria (*n* = 5), serious adverse event (*n* = 1), fulfilling the exclusion criteria of previous stroke (*n* = 1), stomach upset (*n* = 1), withdrawal of the consent (*n* = 116), and withdrawal with unknown reasons (*n* = 113). Finally, 560 (62.6%) were randomly assigned to receive amlodipine 5 mg/benazepril 10 mg one tablet per day (*n* = 282) or benazepril 10 mg/hydrochlorothiazide 12.5 mg one tablet per day (*n* = 278). A total of 213 patients in the amlodipine/benazepril and 212 in the benazepril/hydrochlorothiazide group completed the study and had a valid repeat ambulatory blood pressure recording during follow-up and were therefore included in the efficacy analysis. Among these patients, 125 patients in the amlodipine/benazepril and 119 in the benazepril/hydrochlorothiazide group had a valid home blood pressure recording at baseline and 24 weeks of follow-up (Fig. 1). All 560 randomized patients were included in the safety analysis.

Patients were comparable between the two randomization groups in all demographic and clinical characteristics at baseline (Table 1). Clinic systolic/diastolic blood pressure was on average 150.9/92.7 mmHg and 151.2/92.5 mmHg in the amlodipine/benazepril and benazepril/hydrochlorothiazide groups, respectively.

**Table 1:** Characteristics of the randomized patients at baseline

Characteristic	Amlodipine/benazepril (*n* = 213)	Benazepril/hydrochlorothiazide (*n* = 212)	*p* value
Men (*n*, %)	123 (57.8)	130 (61.3)	0.45
Age (years)	54.2 ± 12.0	53.6 ± 11.7	0.64
Body mass index (kg/m^2^)	25.0 ± 3.1	25.2 ± 2.8	0.67
Previous antihypertensive treatment (*n*, %)	71 (33.3)	68 (32.1)	0.78
Angiotensin-converting enzyme inhibitors	14 (6.6)	16 (7.6)	0.70
Angiotensin receptor blockers	18 (8.5)	20 (9.4)	0.72
Calcium-channel blockers	32 (15.0)	27 (12.7)	0.50
Diuretics	5 (2.4)	2 (0.9)	0.26
Others	2 (0.9)	3 (1.4)	0.65
Diabetes mellitus (*n*, %)	9 (4.2)	9 (4.3)	0.99
Chronic kidney disease (*n*, %)	2 (0.9)	4 (1.9)	0.41
Clinic blood pressure (mmHg)			
Systolic	150.9 ± 7.4	151.2 ± 8.8	0.75
Diastolic	92.7 ± 5.4	92.5 ± 5.7	0.68
Clinic pulse rate (beats/min)	75.2 ± 7.4	75.2 ± 7.9	0.96
Ambulatory blood pressure (mmHg)			
24-h systolic	143.5 ± 14.1	142.8 ± 13.4	0.58
24-h diastolic	85.9 ± 8.8	86.1 ± 10.1	0.70
Daytime systolic	148.0 ± 14.9	146.7 ± 14.7	0.34
Daytime diastolic	88.8 ± 9.8	88.6 ± 11.1	0.83
Nighttime systolic	133.8 ± 17.7	133.3 ± 16.8	0.77
Nighttime diastolic	79.4 ± 10.0	79.8 ± 11.2	0.67
Home blood pressure (mmHg)	***n* = 125**	***n* = 119**	
Systolic	148.6 ± 18.7	148.6 ± 8.7	0.95
Diastolic	89.4 ± 6.5	89.2 ± 8.0	0.80

Values are mean ± SD, or number of patients (% of column total). Daytime and nighttime were defined as 08:00 to 18:00 and 23:00 to 05:00, respectively. For definitions of diabetes mellitus and chronic kidney disease, see the “Methods” section

### Study treatment and efficacy of treatment during follow-up

During the study treatment period, the study medication in the amlodipine/benazepril group remained one tablet per day in 276 (97.9%) patients and was up-titrated to two tablets per day in 6 (2.1%) patients. The corresponding numbers in the benazepril/hydrochlorothiazide group were 262 (94.2%) and 16 (5.8%), respectively.

**Fig. 2:**
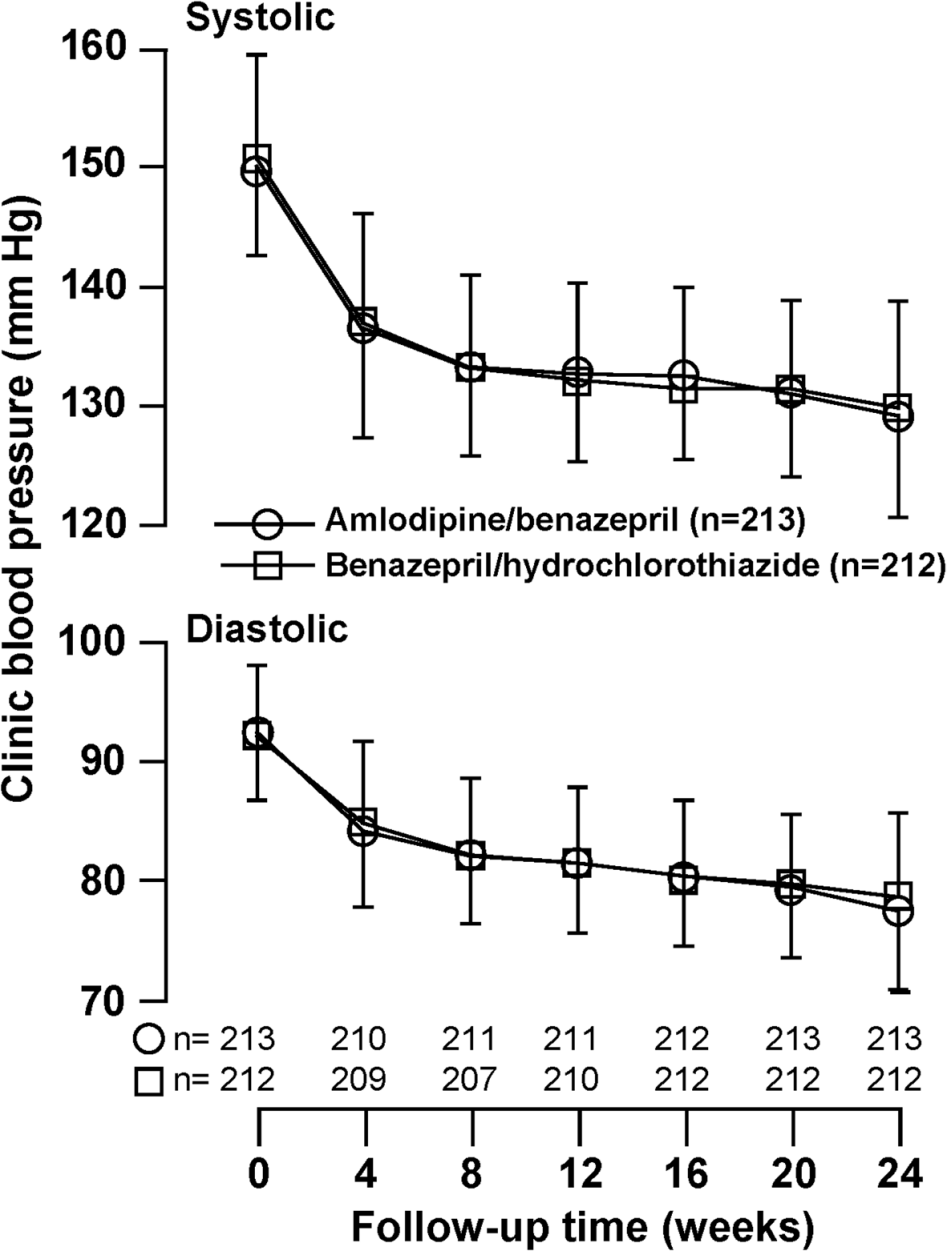
Clinic blood pressure at baseline and during follow-up. Symbols represent the mean values at each clinic visit. Vertical lines denote the standard deviation. The number of patients at each clinic visit is given at the bottom of the figure

During follow-up, clinic systolic/diastolic blood pressure was not significantly (*p* ≥ 0.09) different between the two treatment groups at any clinic visit (Fig. 2). At 24 weeks of follow-up, clinic systolic/diastolic blood pressure was reduced from baseline by − 21.9 ± 0.72/ − 15.3 ± 0.48 mmHg and − 21.4 ± 0.72/ − 13.9 ± 0.48 mmHg in the amlodipine/benazepril (*n* = 213) and benazepril/hydrochlorothiazide groups (*n* = 212), respectively. The corresponding blood pressure control rate was 91.1% and 88.2% (*p* = 0.33), respectively.

**Fig. 3:**
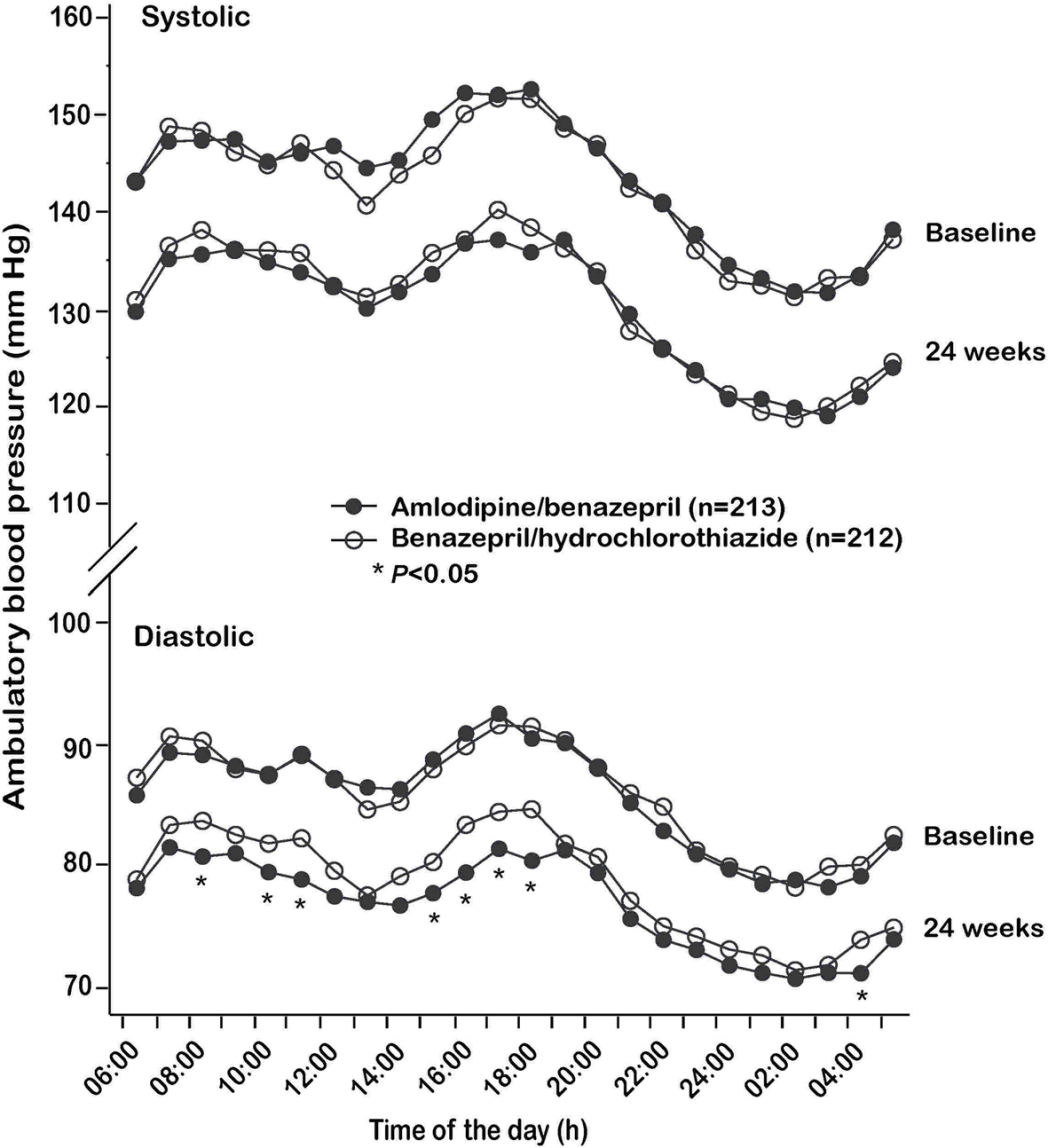
Ambulatory blood pressure at baseline and 24 weeks. Ambulatory systolic and diastolic blood pressure at baseline and after 24 weeks of treatment of amlodipine/benazepril (dot) and benazepril/hydrochlorothiazide (circle) according to time of the day. Asterisks denote *p* value < 0.05 for the difference between the two groups

Twenty-four-hour ambulatory systolic blood pressure change, the primary outcome, was − 13.8 ± 1.17 mmHg (from 143.5 ± 14.1 mmHg at baseline to 129.7 ± 13.7 mmHg at 24 weeks) in the amlodipine/benazepril group and − 12.3 ± 1.17 mmHg (from 142.8 ± 13.4 mmHg at baseline to 130.5 ± 12.7 mmHg at 24 weeks) in the benazepril/hydrochlorothiazide group (Tables 1, 2 and 3). The between-group difference (95% CI) was − 1.51 (− 4.76 to 1.74, *p* = 0.36) mmHg. Among the secondary ambulatory blood pressure outcomes, the between-group differences were not statistically different between the two randomization groups except for the 2.74 mmHg greater reduction in daytime diastolic blood pressure (95% CI, − 5.20 to − 0.27 mmHg, *p* = 0.03) in the amlodipine/benazepril group than in the benazepril/hydrochlorothiazide group (Table 3). GEE analysis by considering the center as a possible bias produced similar results (Additional file 2: Table S1). The ambulatory blood pressure trajectory according to the time of the day showed blood pressures of all hours of the day were comparable at baseline and 24 weeks of follow-up except for diastolic blood pressure at 24 weeks. Diastolic blood pressure in several daytime hours at 24 weeks was significantly lower in the amlodipine/benazepril group than in the benazepril/hydrochlorothiazide group (*p* < 0.05, Fig. 3).

**Table 2:** Clinic, ambulatory, and home blood pressure and clinic pulse rate at 24 weeks

Variable	Amlodipine/benazepril (*n* = 213)	Benazepril/hydrochlorothiazide (*n* = 212)	*p* value
Clinic blood pressure (mmHg)			
Systolic	129.1 ± 8.6	129.8 ± 8.9	0.42
Diastolic	77.4 ± 6.6	78.5 ± 7.0	0.09
Pulse rate (beats/min)	72.2 ± 6.8	71.9 ± 5.9	0.82
Ambulatory blood pressure (mmHg)			
24-h systolic	129.7 ± 13.7	130.5 ± 12.7	0.55
24-h diastolic	76.8 ± 8.7	78.7 ± 9.1	0.04
Daytime systolic	133.8 ± 15.3	135.3 ± 14.2	0.30
Daytime diastolic	78.9 ± 9.6	81.5 ± 10.3	0.01
Nighttime systolic	120.7 ± 15.3	120.7 ± 15.1	0.97
Nighttime diastolic	71.5 ± 9.1	72.9 ± 9.6	0.13
Home blood pressure (mmHg)	***n* = 125**	***n* = 119**	
Systolic	129.9 ± 8.9	130.2 ± 7.6	0.76
Diastolic	77.0 ± 6.6	77.3 ± 6.5	0.72

Daytime and nighttime were defined as 08:00 to 18:00 and 23:00 to 05:00, respectively

**Table 3:** Primary and secondary outcomes between the two treatment groups

Outcomes	Amlodipine/benazepril (*n* = 213)	Benazepril/hydrochlorothiazide (*n* = 212)	Differences (95% CI)	*p* value
**Primary outcome**				
24-h systolic blood pressure change (mmHg)	− 13.8 ± 1.17	− 12.3 ± 1.17	− 1.51 (− 4.76 to 1.74)	0.36
**Secondary outcomes**				
Clinic blood pressure change (mmHg)				
Systolic	− 21.9 ± 0.72	− 21.4 ± 0.72	− 0.44 (− 2.43 to 1.56)	0.67
Diastolic	− 15.3 ± 0.48	− 13.9 ± 0.48	− 1.34 (− 2.68 to 0.00)	0.05
Ambulatory blood pressure change (mmHg)				
24-h diastolic	− 8.95 ± 0.77	− 7.49 ± 0.77	− 1.47 (− 3.60 to 0.67)	0.18
Daytime systolic	− 14.2 ± 1.33	− 11.4 ± 1.33	− 2.86 (− 6.55 to 0.84)	0.13
Daytime diastolic	− 9.83 ± 0.89	− 7.10 ± 0.89	− 2.74 (− 5.20 to − 0.27)	0.03
Nighttime systolic	− 13.0 ± 1.43	− 12.6 ± 1.43	− 0.45 (− 4.43 to 3.53)	0.82
Nighttime diastolic	− 7.87 ± 0.85	− 6.94 ± 0.85	− 0.93 (− 3.30 to 1.44)	0.44
Home blood pressure change (mmHg)	***n* = 125**	***n* = 119**		
Systolic	− 18.7 ± 0.91	− 18.4 ± 0.94	− 0.39 (− 2.96 to 2.19)	0.77
Diastolic	− 12.4 ± 0.67	− 11.9 ± 0.69	− 0.53 (− 2.42 to 1.36)	0.58

Daytime and nighttime were defined as 08:00 to 18:00 and 23:00 to 05:00, respectively. The changes from baseline were calculated by subtracting the blood pressure values at baseline from those at 24 weeks. The least square mean changes (± standard error) were presented in the table. Negative values indicate blood pressure decrease from baseline. The between-group differences were calculated by subtracting the changes in the benazepril/hydrochlorothiazide group from those in the amlodipine/benazepril group. Negative values indicate a greater blood pressure reduction from baseline in the amlodipine/benazepril group than in the benazepril/hydrochlorothiazide group. Analyses in this table were unadjusted for other covariates

Home blood pressure was measured in a subgroup of patients (*n* = 244) and reduced similarly in the amlodipine/benazepril (*n* = 125) and benazepril/hydrochlorothiazide groups (*n* = 119) at each of the follow-up visits (*p* ≥ 0.30, Table 3).

### Subgroup analyses on the 24-h systolic blood pressure-lowering efficacy

**Fig. 4:**
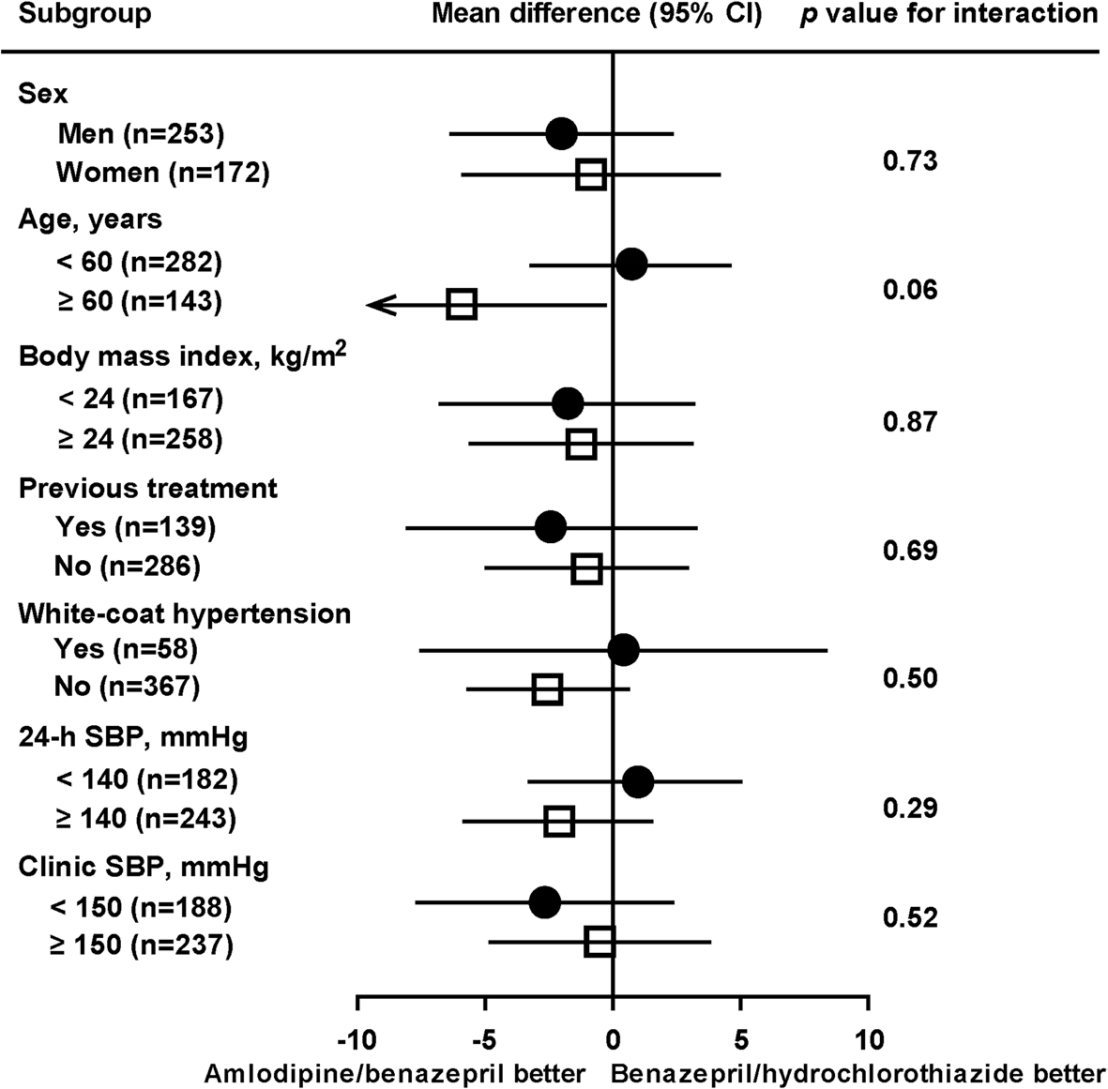
Subgroup analyses on the between-treatment differences in the least square mean changes from baseline in 24-h systolic blood pressure by randomization group. SBP, systolic blood pressure at baseline

We performed subgroup analyses in patients with white-coat (*n* = 58) and sustained hypertension (*n* = 367), in patients who were previously untreated (*n* = 286) and treated with antihypertensive medication (*n* = 139) and in subgroups according to gender, age (≥ 60 vs < 60 years), and clinic (≥ 150 vs < 150 mmHg) and 24-h systolic blood pressure at baseline (≥ 140 vs < 140 mmHg). The interaction was statistically significant in none of these subgroups (*p* ≥ 0.058). Nonetheless, the 24-h systolic blood pressure reduction was significantly greater in the amlodipine/benazepril group than in the benazepril/hydrochlorothiazide group in patients aged ≥ 60 years (− 5.91 mmHg; 95% CI, − 11.8 to − 0.04 mmHg; *p* = 0.048, Fig. 4).

### Safety

One serious adverse event of cerebral hemorrhage occurred during the run-in period of the trial. This patient was hospitalized for 22 days and discharged with stable vital signs. The event was not related to the use of benazepril. The patient was not randomized into the study treatment phase.

During the randomized treatment period, among all 560 randomized patients included in the safety analysis, no serious adverse events were reported, and adverse events were reported in 109 (38.7%) and 124 (44.6%) patients in the amlodipine/benazepril and benazepril/hydrochlorothiazide groups, respectively (*p* = 0.15). The incidence rate of dry cough was significantly lower in the amlodipine/benazepril group than in the benazepril/hydrochlorothiazide group (5.3% vs 10.1%, *p* = 0.04, Table 4). At 24 weeks of follow-up, serum potassium concentration was significantly (*p* < 0.001) lower in the benazepril/hydrochlorothiazide group than in the amlodipine/benazepril group (4.04 ± 0.34 vs 4.17 ± 0.37 mmol/L, *p* < 0.001, Additional file 3: Table S2), with a significant difference between the two groups in the changes from baseline (0.10 mmol/L; 95% CI, 0.03 to 0.17 mmol/L; *p* = 0.006, Additional file 4: Table S3). The incidence rate of hypokalemia, however, was only slightly and non-significantly (*p* = 0.65) higher in the benazepril/hydrochlorothiazide group than in the amlodipine/benazepril group (12.6 vs 11.4%, Table 4). The prevalence of dyslipidemia changed from 64.8% at baseline to 48.9% at 24 weeks of follow-up in the amlodipine/benazepril group and from 58.0% at baseline to 57.4% at 24 weeks of follow-up in the benazepril/hydrochlorothiazide group, with no between-group difference at baseline (*p* = 0.10) and a significant difference at 24 weeks of follow-up (*p* = 0.04, Additional file 3: Table S2). During follow-up, the use of lipid-lowering drugs was reported in four patients (3 in the amlodipine/benazepril group and 1 in the benazepril/hydrochlorothiazide group), and the change in serum total cholesterol or serum triglycerides from baseline between the two groups was not statistically significant (*p* ≥ 0.76, Additional file 4: Table S3).

**Table 4:** Number and incidence rate of adverse events

Adverse event	Amlodipine/benazepril (*n* = 282)	Benazepril/hydrochlorothiazide (*n* = 278)	*p* value
Dizziness	39 (13.8%)	34 (12.2%)	0.57
Hyperuricemia	33 (11.7%)	45 (16.2%)	0.13
Hypokalemia	32 (11.4%)	35 (12.6%)	0.65
Dry cough	15 (5.3%)	28 (10.1%)	0.04
Elevation of alanine or aspartate transaminase	1 (0.4%)	2 (0.7%)	0.55
Ankle edema	1 (0.4%)	0 (0)	0.32
Palpitation	1 (0.4%)	0 (0)	0.32
Stomachache	1 (0.4%)	0 (0)	0.32
Hypotension	1 (0.4%)	0 (0)	0.32
Skin rash	0 (0)	1 (0.4%)	0.31
Hair loss	0 (0)	1 (0.4%)	0.31
Total number of patients with at least one adverse event	109 (38.7%)	124 (44.6%)	0.15

Values are number of patients (% of column total), listed in the descending order of the incidence rate in the amlodipine/benazepril group and then the benazepril/hydrochlorothiazide group

## Discussion

The main finding of our study is that in the Chinese patients with stage 1 to 2 hypertension the amlodipine/benazepril and benazepril/hydrochlorothiazide combinations, both in a single pill, were comparable in ambulatory systolic blood pressure lowering. Nonetheless, these two dual therapies had some differences in ambulatory daytime diastolic blood pressure lowering and in the incidence of dry cough in favor of the amlodipine/benazepril combination. These differences might be clinically relevant for the prevention of cardiovascular events, because 2 to 3 mmHg higher daytime diastolic blood pressure might be associated with 4 to 6% potential risks of cardiovascular events [22] and because the lower incidence of dry cough might be associated with improved treatment adherence.

Our observation on the blood pressure-lowering efficacy is in keeping with the results of the ACCOMPLISH trial [20]. The between-group difference in clinic blood pressure after dose adjustment at 1 month was 0.9 mmHg systolic and 1.1 mmHg diastolic in favor of the amlodipine/benazepril combination. However, the ambulatory blood pressure monitoring study in a subset of patients (*n* = 573) did not show any significant difference in 24-h and daytime or nighttime blood pressure at 2 years between the amlodipine/benazepril and benazepril/hydrochlorothiazide groups. If anything, there were between-group differences of 1.6 mmHg, 1.8 mmHg, and 1.2 mmHg for the mean 24-h, daytime, and nighttime readings in favor of the benazepril/hydrochlorothiazide group [23]. The results, however, might have been confounded by the addition of other antihypertensive drugs. Nonetheless, it is also possible that the blood pressure-lowering efficacy and safety of these dual therapies are ethnicity-dependent.

Ethnicity might also explain why the amlodipine combination with an ACE inhibitor or a thiazide diuretic was more efficacious than a combination of an ACE inhibitor with a thiazide diuretic in the Comparisons of Three Combinations Therapies in Lowering Blood Pressure in Black Africans trial (CREOLE), which was a randomized, single-blind, three-group trial in 621 Black patients with uncontrolled hypertension (≥ 140/90 mmHg while the patient was not being treated or was taking only one antihypertensive drug), recruited from six sub-Saharan African countries [18]. Twenty-four-hour ambulatory systolic blood pressure was significantly lower in the amlodipine/perindopril 5/4 mg and amlodipine/hydrochlorothiazide 5/12.5 mg groups than in the perindopril/hydrochlorothiazide 4/12.5 mg group by − 3.00 mmHg (95% CI, − 5.8 to − 0.20; *p* = 0.04) and − 3.14 mmHg (95% CI, − 5.9 to − 0.38; *p* = 0.03), respectively. In these Black patients with hypertension, the corresponding incidence rate of dry cough was 5.8%, 0%, and 5.0%, respectively,^18^ which was similar to that was observed in the amlodipine/benazepril group of our study (5.3%) but lower than that was observed in the benazepril/hydrochlorothiazide group of our study (10.1%).

Despite that daytime and nighttime ambulatory blood pressure changes were secondary outcomes, the slightly greater reduction in daytime but not nighttime diastolic blood pressure in the amlodipine/benazepril group than in the benazepril/hydrochlorothiazide group is noteworthy. There is abundant evidence that the Chinese have high salt intake [24, 25] and high nighttime blood pressure [26–29]. Diastolic blood pressure, especially on nighttime ambulatory monitoring, is dependent on sodium intake and salt sensitivity [30, 31]. Diuretics are probably more efficacious than other classes of antihypertensive drugs, such as CCBs, in lowering diastolic blood pressure, especially during nighttime sleeping hours [32]. Such a mechanism probably explains why the amlodipine/benazepril combination only showed a greater effect for daytime but not nighttime ambulatory blood pressure lowering.

It is well-known that the Chinese and other Eastern Asians have a higher incidence rate of dry cough associated with the use of ACE inhibitors than other ethnic groups. In a meta-analysis of randomized controlled trials, we previously found that the incidence of dry cough was 10.6% in patients treated with benazepril monotherapy or combination therapy [33]. The mechanisms for the dry cough and ethnic differences remain unclear. It has been postulated that ACE inhibitors inhibit the degradation of bradykinin and substance P by ACE and lead to the accumulation of these peptides in the upper and lower respiratory tracts. Bradykinin induces sensitization of airway sensory nerves via rapidly adapting stretch receptors and C-fiber receptors that release neurokinin A and substance P. This causes airway smooth muscle constriction and leads to bronchoconstriction and cough [15]. There is emerging evidence that combination of an ACE inhibitor with a CCB had an infrequent incidence of dry cough associated with the use of ACE inhibitors [34, 35]. In addition, in an experimental study in porcine ciliary arteries, the sensitivity (concentration shift 73-fold; *p* < 0.05) and maximal relaxation to bradykinin (by 27%; *p* < 0.01) was enhanced by benazeprilat but not amlodipine or the combination of benazeprilat with amlodipine [36].

Our study should be interpreted within the context of its strengths and limitations. Two single-pill combinations were used in the trial. Repeat ambulatory blood pressure recording was performed during follow-up. Our study had an extended 24-week follow-up, which was important not only for the investigation of efficacy and safety, but also for the patients’ acceptance of a repeated ambulatory blood pressure monitoring within months. However, our study had an open design and hence was prone to observers’ and patients’ bias. It could not prove the superiority of SPC to monotherapy, because of the lack of an antihypertensive monotherapy group. Ambulatory blood pressure was not a criterion for inclusion in the trial. We therefore had to rely on the ambulatory blood pressure changes instead of values for the outcome evaluation. In addition, some patients with white-coat hypertension were enrolled in the trial. However, subgroup analyses in patients with white-coat and sustained hypertension did not show significant interaction. The results in an analysis restricting sustained hypertension were not materially different (data not shown). Finally, a proportion of patients did not complete the ambulatory blood pressure monitoring at 24 weeks of follow-up.

## Conclusions

The amlodipine/benazepril and benazepril/hydrochlorothiazide combination therapies were comparable in 24-h systolic blood pressure lowering. Nonetheless, the former, compared with the latter, combination had a slightly greater blood pressure-lowering effect in older patients and on daytime ambulatory monitoring and had a lower incidence rate of dry cough associated with the use of ACE inhibitors.

### Supplementary Information

**Additional file 1. **Protocol.

**Additional file 2: Table S1. **Primary and secondary outcomes between treatment groups with generalized estimating equation analysis.

**Additional file 3: Table S2. **Blood biochemistry measurements at baseline and 24 weeks (*n*=560).

**Additional file 4: Table S3. **Least square mean changes from baseline to 24 weeks of follow-up and between-treatment differences (95% confidence interval) in blood biochemistry measurements (n=560).

**Additional file 5. **The participating hospitals.

## Data Availability

Anonymized data can be made available to the investigators for targeted research based on a motivated request to be addressed to the corresponding author via jiguangwang@rjh.com.cn.

## Abbreviations

ACCOMPLISH: Avoiding Cardiovascular Events through Combination Therapy in Patients Living with Systolic Hypertension
ACE: Angiotensin-converting enzyme
ACvAD: ACEI/CCB versus ACEI/DIU combination antihypertensive therapy in Chinese hypertensive patients
ASCOT: Anglo-Scandinavian Cardiac Outcome Trial
BP: Blood pressure
CCB: Calcium-channel blocker
CI: Confidence interval
CKD-EPI: Chronic Kidney Disease Epidemiology Collaboration
eGFR: Estimated glomerular filtration rate
GEE: Generalized estimating equation
ISH: International Society of Hypertension
SPC: Single-pill combination

## References

[1] Mancia G, Kreutz R, Brunström M, Burnier M, Grassi G, Januszewicz A (2023). 2023 ESH Guidelines for the management of arterial hypertension the task force for the management of arterial hypertension of the European Society of Hypertension Endorsed by the International Society of Hypertension (ISH) and the European Renal Association (ERA). J Hypertens.

[2] Whelton PK, Carey RM, Aronow WS, Casey DE, Collins KJ, Dennison Himmelfarb C (2017). ACC/AHA/AAPA/ABC/ACPM/AGS/APhA/ASH/ASPC/NMA/PCNA guideline for prevention, detection, evaluation, and management of high blood pressure in adults: a report of the American College of Cardiology/American Heart Association Task Force on Clinical Practice Guidelines. J Am Coll Cardiol..

[3] Unger T, Borghi C, Charchar F, Khan NA, Poulter NR, Prabhakaran D (2020). 2020 International Society of Hypertension Global Hypertension Practice Guidelines. Hypertension.

[4] Joint Committee for Guideline Revision (2019). 2018 Chinese guidelines for prevention and treatment of hypertension—a report of the Revision Committee of Chinese guidelines for prevention and treatment of hypertension. J Geriatr Cardiol.

[5] Wang JG, Liu LS (2018). Global impact of 2017 American College of Cardiology/American Heart Association hypertension guideline: a perspective from China. Circulation.

[6] Rea F, Corrao G, Merlino L, Mancia G (2018). Early cardiovascular protection by initial two-drug fixed-dose combination treatment vs. monotherapy in hypertension. Eur Heart J.

[7] Visco V, Finelli R, Pascale AV, Giannotti R, Fabbricatore D, Ragosa N (2017). Larger blood pressure reduction by fixed-dose compared to free dose combination therapy of ACE inhibitor and calcium antagonist in hypertensive patients. Transl Med UniSa.

[8] Ngo L, Cho HY, Lee YB (2018). Effects of hydrochlorothiazide and amlodipine on single oral dose pharmacokinetics of valsartan in healthy Korean subjects: population model-based approach. Eur J Pharm Sci.

[9] Yusuf S, Teo KK, Pogue J, Dyal L, Copland I, ONTARGET Investigators (2008). Telmisartan, ramipril, or both in patients at high risk vascular events. N Engl J Med.

[10] Godfraind T (2014). Calcium channel blockers in cardiovascular pharmacotherapy. J Cardiovasc Pharmacol Ther.

[11] Musini VM, Nazer M, Bassett K, Wright JM (2014). Blood pressure-lowering efficacy of monotherapy with thiazide diuretics for primary hypertension. Cochrane Database Syst Rev.

[12] Liang L, Kung JY, Mitchelmore B, Gill J, Cave A, Banh HL (2021). Angiotensin-converting enzyme inhibitor induced cough in Chinese patients: a systematic review and meta-analysis. J Pharm Pharm Sci.

[13] Antza C, Stabouli S, Kotsis V (2016). Combination therapy with lercanidipine and enalapril in the management of the hypertensive patient: an update of the evidence. Vasc Health Risk Manag.

[14] Borghi C, Soldati M, Bragagni A, Cicero AFG (2020). Safety implications of combining ACE inhibitors with thiazides for the treatment of hypertensive patients. Expert Opin Drug Saf.

[15] Pinto B, Jadhav U, Singhai P, Sadhanandham S, Shah N (2020). ACEI-induced cough: a review of current evidence and its practical implications for optimal CV risk reduction. Indian Heart J.

[16] Weinstein J, Girard LP, Lepage S, McKelvie RS, Tennankore K (2021). Prevention and management of hyperkalemia in patients treated with renin-angiotensin-aldosterone system inhibitors. Can Med Ass J.

[17] Clase CM, Carrero JJ, Ellison DH, Grams ME, Hemmelgam BR, Jardine MJ (2020). Potassium homeostasis and management of dyskalemia in kidney diseases: conclusions from a Kidney Disease: Improving Global Outcomes (KDIGO) Controversies Conference. Kidney Int.

[18] Ojji DB, Mayosi B, Francis V, Badri M, Cornelius V, Smythe W (2019). Comparison of dual therapies for lowering blood pressure in black Africans. N Engl J Med.

[19] Dahlöf B, Sever PS, Poulter NR, Wedel H, Beevers DG, Caulfield M (2005). Prevention of cardiovascular events with an antihypertensive regimen of amlodipine adding perindopril as required versus atenolol adding bendrofluethiazide as required, in the Anglo-Scandinavian Cardiac Outcomes Trial-Blood Pressure Lowering Arm (ASCOT-BPLA): a multicentre randomised controlled trial. Lancet.

[20] Jamerson K, Weber MA, Bakris GL, Dahlöf B, Pitt B, Shi V (2008). Benazepril plus amlodipine or hydrochlorothiazide for hypertension in high-risk patients. N Engl J Med.

[21] Levey AS, Stevens LA, Schmid CH, Zhang Y, Castro AF, Feldman HI (2009). A new equation to estimate glomerular filtration rate. Ann Intern Med.

[22] Yang WY, Melgarejo JD, Thijs L, Zhang ZY, Boggia J, Wei FF (2019). Association of office and ambulatory blood pressure with mortality and cardiovascular outcomes. JAMA.

[23] Jamerson KA, Devereux R, Bakris GL, Dahlöf B, Pitt B, Velazquez EJ (2011). Efficacy and duration of benazepril plus amlodipine or hydrochlorthiazide on 24-hour ambulatory systolic blood pressure control. Hypertension.

[24] Fan F, Li Y, Li L, Nie X, Zhang P, Li Y (2022). Salt-related knowledge, attitudes, and behaviors and their relationship with 24-hour urinary sodium excretion in Chinese adults. Nutrients.

[25] Okuda N, Stamler J, Brown IJ, Ueshima H, Miura K, Okayama A (2022). Individual efforts to reduce salt intake in China, Japan, UK, USA: what did people achieve? The INTERMAP Population Study. J Hypertens.

[26] Li Y, Wang JG, Gao HF, Nawrot T, Wang GL, Qian YS (2005). Are published characteristics of the ambulatory blood pressure generalizable to rural Chinese? The JingNing population study. Blood Press Monit.

[27] Li Y, Staessen JA, Lu L, Li LH, Wang GL, Wang JG (2007). Is isolated nocturnal hypertension a new clinical entity?. Hypertension.

[28] Li Y, Wang JG (2013). Isolated nocturnal hypertension: a disease masked in the dark. Hypertension.

[29] Fan HQ, Li Y, Thijs L, Hansen TW, Boggia J, Kikuya M (2010). Prognostic value of isolated nocturnal hypertension on ambulatory measurement in 8711 individuals from 10 populations. J Hypertens.

[30] Zou J, Li Y, Yan CH, Wei FF, Zhang L, Wang JG (2013). Blood pressure in relation to interactions between sodium dietary intake and renal handling. Hypertension.

[31] Kang YY, Cheng YB, Guo QH, Sheng CS, Huang QF, Xu TY (2021). Renal sodium handling in relation to environmental and genetic factors in untreated Chinese. Am J Hypertens.

[32] Fujiwara T, Hoshide S, Tomitani N, Kanegae H, Kario K (2021). Comparative effects of valsartan plus cilnidipine or hydrochlorothiazide on nocturnal home blood pressure. J Clin Hypertens.

[33] Wang JG, Xie LD, Zhan SY, on behalf of the EVIDENCE CHINA Study Group (2011). The antihypertensive efficacy and safety of benazepril in Chinese population: a meta-analysis of randomized controlled trials. Chin J Hypertens.

[34] Laurent S, Parati G, Chazova I, Sirenko Y, Erglis A, Laucevicius A (2015). Randomized evaluation of a novel, fixed-dose combination of perindopril 3.5 mg/amlodipine 2.5 mg as a first-step treatment in hypertension. J Hypertens.

[35] Mourad JJ, Amodeo C, de Champvallins M, Brzozowska-Villatte R, Asmar R, on behalf of the study coordinators and investigators (2017). Blood pressure-lowering efficacy and safety of perindopril/indapamide/amlodipine single-pill combination in patients with uncontrolled essential hypertension: a multicenter, randomized, double-blind, controlled trial. J Hypertens.

[36] Lang MG, Zhu P, Meyer P, Noll G, Haefliger IO, Flammer J (1997). Amlodipine and benazeprillat differently affect the responses to endothelin-1 and bradykinin in porcine ciliary arteries: effects of a low and high dose combination. Curr Eye Res.

