# Supplementary material for: Comparison of two single-pill dual combination antihypertensive therapies in Chinese patients: a randomized, controlled trial
Source: BMC Med. 2024 Jan 24;22:28. doi: 10.1186/s12916-023-03244-4 (PMC10807184; doi:10.1186/s12916-023-03244-4)
Supplement: Supplementary file 1 — Additional file 1. Protocol. [file 12916_2023_3244_MOESM1_ESM.pdf]

Protocol

(Study Description in Consistence with the Chinese Protocol Version 2, Version date October 22nd, 2018)

ACEI/CCB versus ACEI/DIU combination antihypertensive therapy

in Chinese hypertensive patients

(ACvAD)

-A Randomized Controlled Trial-

Principal Investigator:

Ji-Guang Wang, Yan Li

Centre for Epidemiological Studies and Clinical Trials, Ruijin Hospital

Contact Address:

Shanghai Institute of Hypertension, Ruijin 2nd Road 197, Shanghai 200025, China.

Tel: +86 21 6437 0045 ext. 610911

Fax: +86 21 6431 3816

Email: jiguangwang@rjh.com.cn

Rationale

A meta-analysis of 42 studies showed that the combination of two antihypertensive drugs is better than monotherapy to control blood pressure [1]. Most patients with grade 2 or higher hypertension need two or more antihypertensive drugs to control blood pressure. Antihypertensive therapy with fixed-dose combination can better control blood pressure and improve patient compliance, as well as reduce adverse effect. For untreated patients with high blood pressure level, the guidelines recommend that combination therapy can be used initially. The fixed-dose combination can control blood pressure to target, as well as improve patient compliance by reducing the number of tablets [2].

Most current hypertension guidelines, including the Chinese guidelines for the management of hypertension, recommend the combination therapy of an angiotensin-converting enzyme inhibitors (ACEI) or angiotensin receptor blockers (ARB) with a calcium antagonists (CCB) or thiazide diuretics (DIU) [2,3]. CHIEF (Chinese Hypertension Intervention Efficacy study) showed that long-term combination therapy with amlodipine plus telmisartan or amlodipine plus amiloride/hydrochlorothiazide was not only well tolerated but also efficacious in reducing BP levels with acceptable control rates in the majority of hypertensive patients [4]. ACCOMPLISH (The Avoiding Cardiovascular Events through Combination Therapy in Patients Living with Systolic Hypertension) trial reported that blood pressure control, which was defined as a blood pressure of less than 140/90 mm Hg, was attained in an average of 75.4% of patients in the benazepril–amlodipine group and 72.4% in the benazepril–hydrochlorothiazide group [5]. However, whether the two combination treatments have similar antihypertensive effects in Chinese hypertensive patients is still lack of clinical evidence, especially randomized controlled trials using ambulatory or home blood pressure measurement as the main evaluation method.

24-h ambulatory blood pressure is more predictive of cardiovascular events than clinic pressure measured in doctor’s office. Several previous studies have demonstrated that an exaggerated morning blood pressure surge is associated with the incidence of stoke[6,7] and cardiovascular events [8,9]. Ambulatory blood pressure monitoring and home blood pressure monitoring help blood pressure management [10]. Moreover, non-dipping of blood pressure at night (non-dipper, nighttime blood pressure fall less than 10%) is prevalent and is associated with a higher risk of cardiovascular event [11]. In our JingNing population study, we found that Chinese had a higher nighttime diastolic blood pressure than White populations, and the prevalence of

isolated nocturnal hypertension was 10.9% [12]. Patients with isolated nocturnal hypertension compared with normotensive subjects had an increased arterial stiffness [13]. Hypertension classified according to 24-h systolic or diastolic blood pressure showed prognostic value and age difference [14]. Thus, a preferable antihypertensive drug should not only reduce the office blood pressure, but should also effectively control 24-h systolic and diastolic blood pressure as well as morning and nighttime blood pressure.

This randomized controlled clinical trial will be carried out in 580 Chinese hypertensive patients. By measuring office blood pressure, 24-hour ambulatory blood pressure and home blood pressure, the blood pressure lowering effect of benazepril 10 mg/amlodipine besylate 5 mg fixed-dose combination (ACEI/CCB) and benazepril 10 mg/hydrochlorothiazide 12.5 mg fixed-dose combination (ACEI/DIU) will be compared.

Objective

The primary objective is to evaluate the reduction of 24-hour ambulatory systolic blood pressure in the ACEI/CCB group compared with the ACEI/DIU group after 6 months of antihypertensive treatment in grade 1-2 hypertensive patients with the age over 18 years, either newly diagnosed or monotherapy not controlled.

The secondary objectives are to compare the reduction of 24-hour, daytime, night-time, home and office systolic and diastolic blood pressure as well as blood biochemistry measurements, such as fasting plasma glucose, serum triglycerides and cholesterol, electrolyte, creatinine and uric acid, etc., after 6 months of study medication treatment.

Study design

This study is a multi-center, randomized and controlled clinical trial (Phase IV) with two equally sized treatment groups: ACEI/CCB group and ACEI/DIU group (Figure).

Figure: Study design.

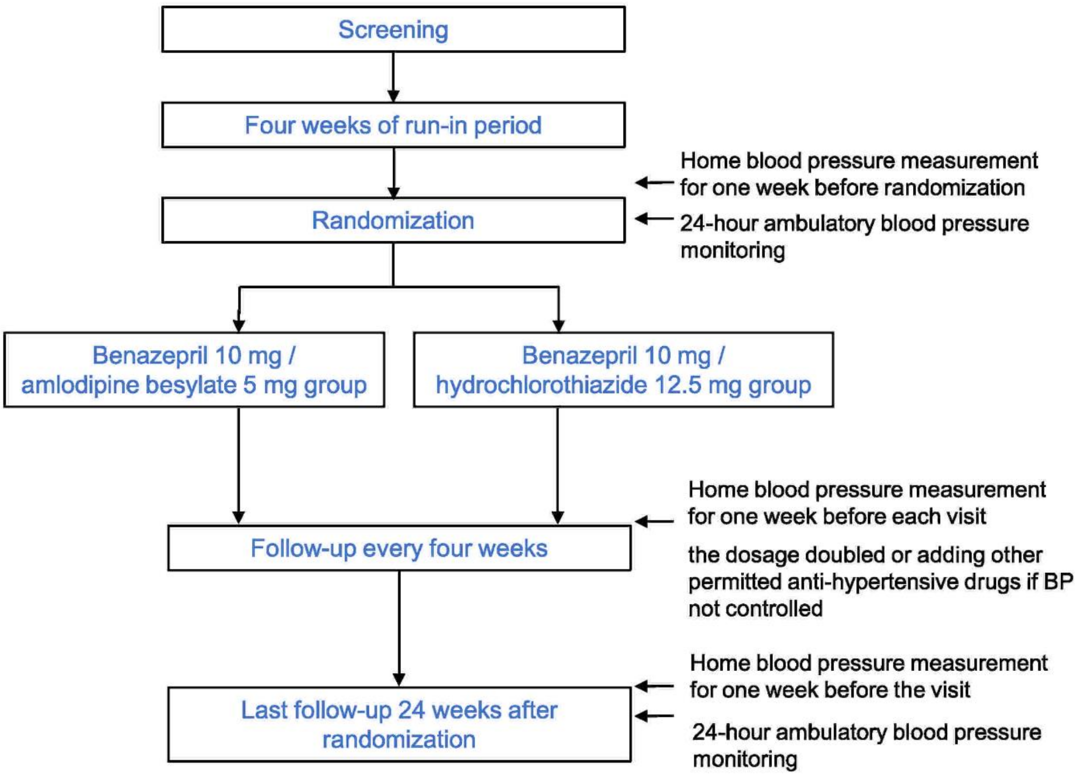

Primary and secondary efficacy variables

The primary efficacy variable was the change from baseline to 24 weeks of treatment in 24-h ambulatory systolic blood pressure.

Secondary efficacy variables included,

1. the change from baseline to 24 weeks of treatment in 24-h ambulatory diastolic blood pressure, daytime and nighttime systolic and diastolic blood pressure, and clinic and home systolic and diastolic blood pressure;
2. changes from baseline to one, two, three, four and five months in home and office systolic and diastolic blood pressure;
3. changes from baseline to 24 weeks of treatment in fasting plasma glucose, serum lipid (triglycerides, total cholesterol, high density lipoprotein cholesterol and low density lipoprotein cholesterol), serum electrolyte (potassium, sodium and chlorine), serum creatinine and serum uric acid.

Study population

580 eligible patients are anticipated to be randomized into this study in 15 to 20

hospitals and community health centers across China.

Inclusion criteria:

1. the age over 18 years;
2. newly diagnosed grade 1-2 hypertensive patients or grade 1-2 hypertensive patients with monotherapy whose blood pressure not controlled, having systolic blood pressure ranged from 140 to 179 mmHg or (and) diastolic blood pressure ranged from 90 to 109 mmHg;
3. can enter a 4-week run-in period before randomization. During the run-in period, all patients take benazepril 10 mg daily, and have an office visit every two weeks. After taking benazepril for 4 weeks continually with no adverse effect, patients with the mean office blood pressure of the last two visits between 140 and 179 mmHg in systolic or (and) between 90 and 109 mmHg in diastolic are eligible for randomization. Eligible patients will switch to take study medication after randomization;
4. A one-week home blood pressure measurement should be performed before randomization by using an automatic device;
5. Patient should agree to participate in the trial, and can pay follow-up visits.

Exclusion criteria:

1. suspected or confirmed secondary hypertension;
2. history of coronary heart disease, myocardial infarction, heart failure, stroke or dementia;
3. other drugs that might affect blood pressure;
4. serum levels of ALT, AST, TBL equal or higher than twice of the upper limit;
5. serum creatinine ≥1.5 mg/dL (133 μmol/L);
6. urine protein positive;
7. serum potassium >5.5 mmol/L or <3.5 mmol/L;
8. history of gout or serum uric acid ≥420 μmol/L for male or ≥360 μmol/L for female;
9. elderly patients need caring;
10. patients who are participating other clinical trials.

Randomization and treatment

Potentially eligible patients should receive a 24-hour ambulatory blood pressure monitoring measurement before randomization. After stratification by centers and whether receive antihypertensive treatment, eligible patients will be randomly divided into two groups, taking benazepril 10 mg/amlodipine besylate 5 mg fixed-dose combination (1 tablet once a day) or benazepril 10 mg/ hydrochlorothiazide 12.5 mg fixed-dose combination (1 tablet once a day). If the systolic/diastolic blood pressure higher than 140/90 mmHg during follow-up visits, the dosage can be increased to 2 tablets once a day. If the systolic/diastolic blood pressure still ≥140/90 mmHg, bisoprolol 5 mg (1 tablet once a day) or doxazosin controlled-release tablets 4 mg (1 tablet once a day) or spironolactone 20 mg (1 tablet once a day) can be added during the following follow-up visits. To compare the efficacy accurately, all study medications should be taken between 6 am to 8 am every morning before breakfast.

Follow up

All patients should sign an informed consent form before entering the study. A validated Omron automatic device should be used to measure the sitting blood pressure in the clinic. Patients will be followed up every month with sitting office blood pressure measured and medical history recorded. Before each follow-up visit, a one-week home blood pressure measurement should be performed using an automatic Omron device; 24-hour ambulatory blood pressure monitoring, electrocardiogram, blood and urine biochemical tests should be repeated at the end of the 6 months of follow-up.

Office blood pressure should be recorded in detail. Drug usage, office blood pressure, heart rate, serious adverse event, adverse event and the occurrence of cardiovascular diseases should be recorded.

Ambulatory blood pressure monitoring should be performed using a validated oscillometric ambulatory blood pressure monitor, which should be programmed to obtain ambulatory blood pressure readings at 20-minute intervals in the day (06:00-22:00) and at 30-minute intervals at night (22:00-06:00). Patients should be required to report the get up and sleep time on the day of ambulatory blood pressure monitoring, as well as medication usage and physical activity.

Flowchart

Visits	V0	V1	V2	V3	V4	V5	V6	V7	V8
Week	W−4	W-2	W0	W4	W8	W12	W16	W20	W24
Consent	×								
Demography	×								
Inclusion/exclusion criteria	×		×						
Medical history	×								
Physical examination	×	×	×	×	×	×	×	×	×
Blood pressure and heart rate	×	×	×	×	×	×	×	×	×
Accompany treatment	×	×	×	×	×	×	×	×	×
Laboratory examination	× [a]		×	× [b]					×
24-h ambulatory blood pressure monitoring			×						×
Home blood pressure measurement			×	×	×	×	×	×	×
Electrocardiogram			×						×
Adverse event (s)		×	×	×	×	×	×	×	×
Medication distribution	×	×	×	×	×	×	×	×	
Medication counting		×	×	×	×	×	×	×	×

[a]Pregnancy test performed before women of childbearing age enter the run-in period.
[b]Serum potassium, sodium and chlorine measured four weeks after randomization.

Possible problems in the study

During data collection, ambulatory blood pressure monitoring less than 70% of the expected blood pressure readings, less than 20 readings in the daytime (or awake), and less than 7 readings in the night (or sleep) will be regarded as unqualified monitoring. In this case, ambulatory blood pressure monitoring should be repeated.

Sample size estimation

The primary outcome is the reduction of 24-hour ambulatory systolic blood pressure in the ACEI/CCB group compared with the ACEI/DIU group after 6 months of antihypertensive treatment. Assuming the difference between groups is 2.5 mmHg and the standard deviation is 10 mmHg, α is 0.05, and the power is 80%, the sample size of each group should be 252 patients. Accounting for 15% of add-up, each group needs 290 eligible patients. The total number of patients is 580. The data will be analyzed by SAS software, and the t-test will be used to compare the continuous variables of two groups.

Timeline

Ethical review: May 2018; Enrollment: May 2018- April 2019; End of follow-up: October 2019. At the same, abstract of the main results will be submitted to domestic or international scientific conferences. The main results will be published in an international medical journal.

Organization

The principal investigator will be Professor Jiguang Wang and Professor Yan Li from the Centre for Epidemiological Studies and Clinical Trials, Ruijin Hospital, Shanghai, China. 15 to 20 hospitals and community health centers across China will be invited to participate.

References:

1. Wald DS, Law M, Morris JK, Bestwick JP, Wald NJ. Combination therapy versus monotherapy in reducing blood pressure: meta-analysis on 11,000 participants from 42 trials. Am J Med 2009; 122: 290-300.

2. Mancia G, Fagard R, Narkiewicz K, Redon J, Zanchetti A, Bohm M et al. 2013 ESH/ESC guidelines for the management of arterial hypertension: the Task Force for

the Management of Arterial Hypertension of the European Society of Hypertension (ESH) and of the European Society of Cardiology (ESC). Eur Heart J 2013; 34: 2159-219.

3. Liu LS, Writing Group of 2010 Chinese Guidelines for the Management of Hypretension. 2010 Chinese guidelines for the management of hypertension. Zhonghua Xin Xue Guan Bing Za Zhi 2011; 39: 579-615.

4. Ma L, Wang W, Zhao Y, Zhang Y, Deng Q, Liu M et al. Combination of amlodipine plus angiotensin receptor blocker or diuretics in high-risk hypertensive patients. Am J Cardiovasc Drugs 2012; 12: 137-42.

5. Jamerson K, Weber MA, Bakris GL, Dahlöf B, Pitt B, Shi V et al. Benazapril plus amlodipine or hydrochlorothiazide for hypertension in high-risk patients. N Engl J Med 2008; 359: 2417-28.

6. Kario K, Pickering TG, Umeda Y, Hoshide S, Hoshide Y, Morinari M et al. Morning surge in blood pressure as predictor of silent and clinical cerebrovascular disease in elderly hypertensives. A prospective study. Circulation 2003; 107: 1401-6.

7. Metoki H, Ohkubo T, Kikuya M, Asayama K, Obara T, Hashimoto J et al. Prognostic significance for stroke of a morning pressor surge and a nocturnal blood pressure decline: the Ohasama study. Hypertension 2006; 47: 149-54.

8. Li Y, Thijs L, Hansen TW, Kikuya M, Boggia J, Richart T et al. Prognostic value of the morning blood pressure surge in 5645 subjects from 8 populations. Hypertension 2010; 55: 1040-8.

9. Kario K, Satito I, Kushiro T, Teramukai S, Ishikawa Y, Mori Y et al. Home blood pressure and cardiovascular outcomes in patients during antihypertensive therapy. Hypertension 2014; 64: 989-96.

10. Wang JG, Kario K, Park JB, Chen CH. Morning blood pressure monitoring in the management of hypertension. J Hypertens 2017; 35: 1554-63.

11. Boggia J, Li Y, Thijs L, Hansen TW, Kikuya M, Björklund-Bodegård K et al. Prognostic accuracy of day versus night ambulatory blood pressure: a cohort study. Lancet 2007; 370: 1219-29.

12. Li Y, Wang JG. Isolated nocturnal hypertension: a disease masked in the dark. Hypertension 2013; 61: 278-83.

13. Li Y, Staessen JA, Lu L, Li LH, Wang GL, Wang JG. Is isolated nocturnal hypertension a novel clinical entity? Findings from a Chinese population study.

Hypertension 2007; 50: 333-9.

14. Li Y, Wei FF, Thijs L, Boggia J, Asayama K, Hansen TW et al. Ambulatory hypertension subtypes and 24-hour systolic and diastolic blood pressure as distinct outcome predictors in 8341 untreated people recruited from 12 populations. Circulation 2014; 130: 466-74.
