# Supplementary material for: Comparison of two single-pill dual combination antihypertensive therapies in Chinese patients: a randomized, controlled trial
Source: BMC Med. 2024 Jan 24;22:28. doi: 10.1186/s12916-023-03244-4 (PMC10807184; doi:10.1186/s12916-023-03244-4)
Supplement: Supplementary file 2 — Additional file 2: Table S1. Primary and secondary outcomes between treatment groups with generalized estimating equation analysis. [file 12916_2023_3244_MOESM2_ESM.docx]

**Additional file 2: Table S1.** Primary and secondary outcomes between treatment groups with generalized estimating equation analysis

| **Outcomes** | **Amlodipine/ benazepril**  **(n = 213)** | **Benazepril/ hydrochlorothiazide**  **(n = 212)** | **Differences  (95% CI)** | ***p* value** |
| --- | --- | --- | --- | --- |
| **Primary outcome** |  |  |  |  |
| 24-h systolic blood pressure change (mmHg) | -13.8±1.17 | -12.3±1.17 | -1.65 (-4.73 to 1.43) | 0.29 |
| **Secondary outcomes** |  |  |  |  |
| Clinic blood pressure change (mmHg) |  |  |  |  |
| Systolic | -21.9±0.72 | -21.4±0.72 | -0.53 (-2.35 to 1.29) | 0.57 |
| Diastolic | -15.3±0.48 | -13.9±0.48 | -1.28 (-2.51 to -0.04) | 0.04 |
| Ambulatory blood pressure change (mmHg) |  |  |  |  |
| 24-h diastolic | -8.95±0.77 | -7.49±0.77 | -1.65 (-3.72 to 0.42) | 0.12 |
| Daytime systolic | -14.2±1.33 | -11.4±1.33 | -2.96 (-6.46 to 0.55) | 0.10 |
| Daytime diastolic | -9.83±0.89 | -7.10±0.89 | -2.93 (-5.30 to -0.55) | 0.02 |
| Nighttime systolic | -13.0±1.43 | -12.6±1.43 | -0.66 (-4.46 to 3.13) | 0.73 |
| Nighttime diastolic | -7.87±0.85 | -6.94±0.85 | -1.14 (-3.43 to 1.15) | 0.33 |
| Home blood pressure change (mmHg) | **n = 125** | **n = 119** | **n = 244** |  |
| Systolic | -18.7±0.91 | -18.4±0.94 | -0.34 (-2.77 to 2.09) | 0.78 |
| Diastolic | -12.4±0.67 | -11.9±0.69 | -0.67 (-2.45 to 1.11) | 0.46 |

Daytime and nighttime were defined as 08:00 to 18:00 and 23:00 to 05:00, respectively. The changes from baseline were calculated by subtracting blood pressure values at baseline from that at 24 weeks. Least square mean changes (±standard error) were presented in the table. Negative values indicate blood pressure decrease from baseline. Generalized estimating equation (GEE) analysis was applied by considering center as possible bias. The between-group differences were calculated by subtracting the changes in the benazepril/hydrochlorothiazide group from that in the amlodipine/benazepril group. Negative values indicate a greater blood pressure reduction from baseline in the amlodipine/benazepril than benazepril/hydrochlorothiazide group. Analyses in this table were unadjusted for other covariates.
