# Supplementary material for: Comparison of two single-pill dual combination antihypertensive therapies in Chinese patients: a randomized, controlled trial
Source: BMC Med. 2024 Jan 24;22:28. doi: 10.1186/s12916-023-03244-4 (PMC10807184; doi:10.1186/s12916-023-03244-4)
Supplement: Supplementary file 3 — Additional file 3: Table S2. Blood biochemistry measurements at baseline and 24 weeks (n=560). [file 12916_2023_3244_MOESM3_ESM.docx]

**Additional file 3: Table S2.** Blood biochemistry measurements at baseline and 24 weeks (n=560)

| **Blood biochemistry** | **Amlodipine/ benazepril**  **(n = 284)** | **Benazepril/ hydrochlorothiazide**  **(n = 276)** | ***p* value** |
| --- | --- | --- | --- |
| At baseline |  |  |  |
| Fasting plasma glucose (mmol/L) | 5.62±1.07 | 5.56±0.76 | 0.47 |
| Serum total cholesterol (mmol/L) | 4.78±1.01 | 4.76±0.99 | 0.81 |
| Serum HDL cholesterol (mmol/L) | 1.34±0.39 | 1.33±0.33 | 0.62 |
| Serum total-to-HDL cholesterol ratio | 3.77±1.09 | 3.78±1.06 | 0.91 |
| Serum triglycerides (mmol/L) | 1.60 (1.10-2.30) | 1.60 (1.10-2.10) | 0.43 |
| Serum potassium (mmol/L) | 4.09±0.37 | 4.06±0.36 | 0.26 |
| Serum uric acid (µmol/L) | 319.6±65.9 | 312.5±67.2 | 0.20 |
| eGFR (mL/min/1.73 m2) | 96.4±15.1 | 95.7±14.3 | 0.58 |
| Dyslipidemia (n, %) | 184 (64.8) | 160 (58.0) | 0.10 |
| High serum total cholesterol (n, %) | 110 (38.7) | 95 (34.4) | 0.29 |
| High serum triglycerides (n, %) | 135 (47.5) | 120 (43.5) | 0.34 |
| At 24 weeks |  |  |  |
| Fasting plasma glucose (mmol/L) | 5.78±1.12 | 5.62±0.75 | 0.06 |
| Serum total cholesterol (mmol/L) | 4.77±1.02 | 4.70±1.01 | 0.46 |
| Serum HDL cholesterol (mmol/L) | 1.39±0.38 | 1.41±0.43 | 0.58 |
| Serum total-to-HDL cholesterol ratio | 3.63±1.09 | 3.52±0.98 | 0.21 |
| Serum triglycerides (mmol/L) | 1.55 (1.10-2.10) | 1.50 (1.10-2.09) | 0.37 |
| Serum potassium (mmol/L) | 4.17±0.37 | 4.04±0.34 | <0.001 |
| Serum uric acid (µmol/L) | 328.9±72.1 | 340.2±81.8 | 0.10 |
| eGFR (mL/min/1.73 m2) | 94.6±16.1 | 95.9±15.5 | 0.35 |
| Dyslipidemia (n, %) | 163 (57.4) | 135 (48.9) | 0.04 |
| High serum total cholesterol (n, %) | 101 (35.6) | 79 (28.6) | 0.08 |
| High serum triglycerides (n, %) | 113 (39.8) | 104 (37.7) | 0.61 |

Values are mean±SD or median (interquartile range). HDL: high density lipoprotein. eGFR: estimated glomerular filtration rate. High serum total cholesterol was defined as serum total cholesterol ≥5.18 mmol/L. High serum triglycerides was defined as serum triglycerides ≥1.70 mmol/L. Dyslipidemia was defined as serum total cholesterol ≥5.18 mmol/L and/or serum triglycerides ≥1.70 mmol/L.
