# Supplementary material for: Comparison of two single-pill dual combination antihypertensive therapies in Chinese patients: a randomized, controlled trial
Source: BMC Med. 2024 Jan 24;22:28. doi: 10.1186/s12916-023-03244-4 (PMC10807184; doi:10.1186/s12916-023-03244-4)
Supplement: Supplementary file 4 — Additional file 4: Table S3. Least square mean changes from baseline to 24 weeks of follow-up and between-treatment differences (95% confidence interval) in blood biochemistry measurements (n=560). [file 12916_2023_3244_MOESM4_ESM.docx]

**Additional file 4: Table S3.** Least square mean changes from baseline to 24 weeks of follow-up and between-treatment differences (95% confidence interval) in blood biochemistry measurements (n=560)

| **Blood biochemistry** | **Amlodipine/benazepril**  **(n = 284)** | **Benazepril/hydrochlorothiazide**  **(n = 276)** | **Between-treatment difference (95% CI)** | ***p* value** |
| --- | --- | --- | --- | --- |
| Fasting plasma glucose (mmol/L) | 0.13±0.07 | 0.07±0.07 | 0.07 (-0.12 to 0.25) | 0.51 |
| Serum total cholesterol (mmol/L) | -0.01±0.06 | -0.03±0.06 | 0.03 (-0.15 to 0.20) | 0.76 |
| Serum HDL cholesterol (mmol/L) | 0.04±0.02 | 0.08±0.02 | -0.03 (-0.10 to 0.03) | 0.32 |
| Serum total-to-HDL cholesterol ratio | -0.12±0.06 | -0.23±0.06 | 0.11 (-0.07 to 0.28) | 0.23 |
| Serum triglycerides (mmol/L) | -0.10±0.10 | -0.13±0.10 | 0.02 (-0.26 to 0.31) | 0.87 |
| Serum potassium (mmol/L) | 0.09±0.03 | -0.01±0.03 | 0.10 (0.03 to 0.17) | 0.006 |
| Serum uric acid (µmol/L) | 9.94±4.85 | 28.9±4.99 | -18.9 (-32.6 to -5.24) | 0.007 |
| eGFR (mL/min/1.73 m2) | -1.70±0.93 | 0.14±0.95 | -1.84 (-4.45 to 0.76) | 0.17 |

Values are mean±SE for the changes from baseline to 24 weeks of treatment, unless indicated otherwise. Changes were calculated by subtracting the values during follow-up from that at baseline. Negative values indicate a reduction from baseline. HDL: high density lipoprotein. eGFR: estimated glomerular filtration rate.
