# Supplementary material for: Comparison of two single-pill dual combination antihypertensive therapies in Chinese patients: a randomized, controlled trial
Source: BMC Med. 2024 Jan 24;22:28. doi: 10.1186/s12916-023-03244-4 (PMC10807184; doi:10.1186/s12916-023-03244-4)
Supplement: Supplementary file 5 — Additional file 5. The participating hospitals. [file 12916_2023_3244_MOESM5_ESM.docx]

**Additional file 5: The participating hospitals:**

Yiwu Beiyuan Central Health Center, Yiwu, Zhejiang Province, China (Kai Zhao, n=190), Chongming Branch of Shanghai Tenth people’s Hospital, Shanghai, China (Yihong Luo, n=62), LongHua Hospital, Shanghai University of Traditional Chinese Medicine, Shanghai, China (Bing Deng, n=34), Ruijin Hospital North, Shanghai Jiaotong University School of Medicine, Shanghai, China (Xin Chen, n=33), Suzhou Hospital of Anhui Medical University (Suzhou Municipal Hospital of Anhui Province), Suzhou, Anhui Province, China (Yinglong Qiu, n=33), Puyang People’s Hospital, Puyang, Henan Province, China (Mei-Ling Wang, n=33), Shanghai Pudong New District Zhoupu Hospital, Shanghai, China (Zhihong Zhou, n=32), Sijing Hospital of Songjiang District, Shanghai, China (Leijun Wang, n=32), Yangpu Hospital, Tongji University, Shanghai, China (Jiahong Wang, n=29), Ruijin Hospital, Shanghai Jiaotong University School of Medicine, Shanghai, China (Yan Li and Ji-Guang Wang, n=18), Luodian Hospital of Baoshan District, Shanghai, China (Guibin He, n=17), Shanghai Punan Hospital of Pudong New District, Shanghai, China (Hong Yu, n=15), Zhengzhou Central Hospital Affiliated to Zhengzhou University, Zhengzhou, Henan Province, China (Ping Wang, n=8), Chongming branch of Xinhua Hospital, Shanghai Jiaotong University School of Medicine, Shanghai, China (Yingmin Lu, n=7), Dachang Branch of Renji Hospital, Shanghai Jiaotong University School of Medicine, Shanghai, China (Yanping Zhang, n=6), Affiliated Hospital of North Sichuan Medical College, Nanchong, Sichuan Province, China (Yong Luo, n=5), Shanghai TCM-Integrated Hospital, Shanghai, China (Jianhua Chen, n=2), Henan Province People’s Hospital, Zhengzhou, Henan Province, China (Min Liu, n=2), The Affiliated Hospital of Southwest Medical University, Luzhou, Sichuan Province, China (Qing Peng, n=1), Changzhou Wujin People’s Hospital, Changzhou, Jiangsu Province, China (Jianqiang Xiao, n=1).
